# The Impact of Femoral Anteversion Correction on Clinical Outcomes in Total Hip Arthroplasty for Adult Developmental Dysplasia of the Hip

**DOI:** 10.3390/jcm14093207

**Published:** 2025-05-06

**Authors:** Ahmet Burak Satılmış, Tolgahan Cengiz, Ahmet Ülker, Tansel Mutlu

**Affiliations:** 1 Department of Orthopedics and Traumatology, Taşköprü State Hospital, 37400 Kastamonu, Turkey; tolgahancengiz@hotmail.com; 2 Department of Orthopedics and Traumatology, Mersin University, 33010 Mersin, Turkey; drahmetulker@gmail.com; 3 Department of Orthopedics and Traumatology, Kocaeli Health and Technology University, 41001 Kocaeli, Turkey; tanselmutlu@yahoo.com

**Keywords:** total hip arthroplasty, developmental dysplasia of the hip, femoral anteversion, subtrochanteric osteotomy, hip stability

## Abstract

**Background:** Total hip arthroplasty (THA) in adult patients with developmental dysplasia of the hip (DDH) presents unique surgical challenges due to altered hip anatomy and biomechanics. One of the most critical factors influencing surgical success is correcting femoral anteversion. This study evaluates the effects of different anteversion corrections (increased, unchanged, and decreased) during THA on clinical outcomes in adult Crowe type 3-4 DDH patients. **Methods:** A retrospective analysis was conducted on 130 patients who underwent THA with anteversion correction between 2008 and 2017 at a tertiary university hospital. Patients were divided into three groups: Group 1 (increased anteversion), Group 2 (unchanged anteversion), and Group 3 (decreased anteversion). Clinical outcomes were assessed using AOFAS and Lysholm scores, Q-angle measurements, and physical examinations, while radiological evaluations were performed to determine implant stability and complications. **Results:** Postoperative assessments show that increased anteversion is associated with higher rates of instability, knee dysfunction, and pain. Group 1 had significantly lower AOFAS scores (*p* < 0.001) and fell due to rotational deformity (25.6%). Group 2 exhibited the best clinical outcomes, with the lowest rates of instability and the highest functional scores. Group 3 had moderate functional improvements but a higher dislocation rate (17.1%) than Group 2. **Conclusions:** Our findings suggest that excessive changes in femoral anteversion during THA, remarkably increasing it, may negatively impact clinical outcomes by causing instability and pain. Preserving the anteversion angle as much as possible is the most favorable approach for optimizing functional recovery and implant stability. Further large-scale, long-term studies are required to confirm these findings and refine surgical strategies for DDH patients undergoing THA.

## 1. Introduction

Untreated developmental dysplasia of the hip (DDH) in adulthood often results in progressive anatomical and biomechanical alterations predisposing patients to secondary osteoarthritis, typically manifesting in the fourth and fifth decades of life [1,2]. These patients usually suffer from reduced mobility and pain due to problems such as joint contractures, differences between extremity lengths, and abductor weakness. A subtrochanteric shortening osteotomy can correct increased femoral anteversion (one of the most essential abnormalities caused by DDH). It may be a requirement during the restoration of the anatomical hip center [3]. Although osteotomy is commonly advised when the anteversion angle exceeds 40°, each case should be evaluated individually. The decision for anteversion correction must consider radiological assessment and intraoperative findings to optimize alignment and hip stability [4,5,6]. Patients should be evaluated individually, and anteversion correction should be performed according to their requirements. During total hip arthroplasty (THA), inserting the femoral prosthesis without correction may result in postoperative dislocation. Failure to establish normal balance may also cause the trochanter major to become squeezed in the pelvic wall, adversely affecting the outcome [7].

The Crowe classification is commonly used to grade the severity of hip dislocation in DDH, with types III and IV indicating high-riding femoral heads. Specifically, type III corresponds to proximal displacement between 75% and 100% of the femoral head height [8]. In comparison, type IV exceeds 100%, often requiring femoral shortening osteotomy due to soft tissue tension and neurovascular risk. Studies with appropriate techniques and good postoperative care show that acetabulum reduction can be achieved without femoral shortening and neurological damage [8,9]. However, hip stabilization is significant for good clinical outcomes, and correcting anteversion with subtrochanteric femoral shortening osteotomy can contribute to hip stability. Thus, proper correction of the rotational deformity, appropriate technique, and good postoperative care are crucial for successful total hip arthroplasty (THA) in patients with Crowe type 3–4 high hip dysplasia. In this study, we aim to evaluate the effects of different anteversion corrections performed during total hip arthroplasty (THA) on clinical outcomes in adult Crowe type 3–4 developmental dysplasia of the hip (DDH) patients and to demonstrate that determining the optimal anteversion angle plays a critical role in terms of surgical success and patient satisfaction.

## 2. Materials and Methods

Between 2008 and 2017, patients who underwent total hip arthroplasty (THA) with anteversion correction at the Orthopedic Clinic of a tertiary university hospital were retrospectively analyzed. The inclusion criteria were as follows: (1) adult patients (age ≥ 18) with Crowe type 3 or 4 DDH undergoing primary THA; (2) no previous ipsilateral THA; and (3) available complete clinical and radiological data for a minimum of 24 months follow-up. The exclusion criteria included the following: (1) previous surgery on the affected hip; (2) co-existing neuromuscular disorders; (3) bilateral procedures; (4) septic arthritis history; and (5) refusal or loss to follow-up. Initially, 179 patients were considered for inclusion. However, 49 patients were excluded for the following reasons: 25 had a previous operation at the same site; 15 declined participation or were lost to follow-up; 8 had concurrent hip problems; and 1 had a diagnosis of osteomyelitis. Thus, the study group comprised 130 patients divided into groups according to anteversion correction (increased, unchanged, and decreased anteversion). Patients were stratified into three groups based on intraoperative changes in femoral anteversion relative to preoperative CT measurements. An increase or decrease greater than 10° defined the ‘increased’ and ‘decreased’ groups, respectively. Changes within ±5° were categorized as ‘unchanged’. These thresholds were determined based on preoperative planning, anatomical constraints, and implant trial alignment. In Group 1, transverse subtrochanteric femoral shortening osteotomy was performed through the posterior hip approach, and the prosthesis was inserted with an increased angle of femoral anteversion. The same procedure was performed for Group 2 without changing the femoral anteversion angle. The third group also underwent the same procedure but with a decreased anteversion angle.

### 2.1. Surgical Technique

**Figure 1:**
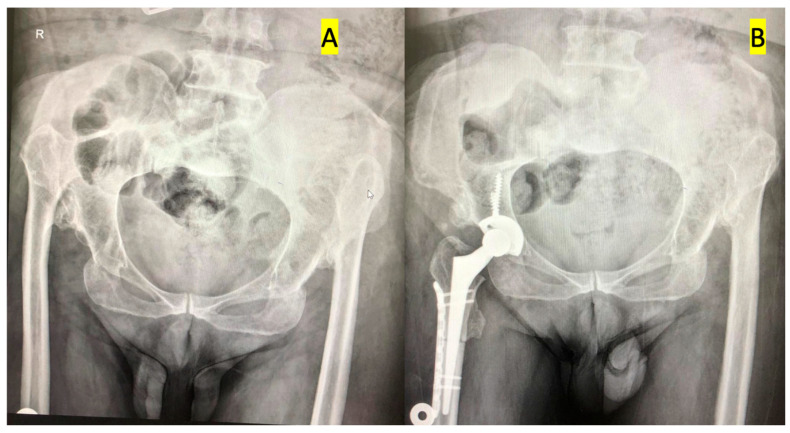
Anteroposterior pelvic radiographs of a Crowe type IV DDH patient. (**A**) Preoperative image showing high dislocation of the femoral head and dysplastic acetabulum. (**B**) Postoperative image after total hip arthroplasty with subtrochanteric shortening osteotomy, demonstrating restored hip center and stable implant fixation.

Percutaneous adductor tenotomy was performed to enable distal relocation of the femur in patients who had limited abduction before surgery. The procedure may be summarized as follows: Patients were placed in the lateral decubitus position on the operating table. The pelvis was supported and splinted from the pubis to the sacrum. A posterolateral incision was used in all patients. The tensor fascia lata was longitudinally cut, the external rotator muscle was suspended by forcing the leg to internal rotation, and finally, the capsule was cut to reach the bone. The femur neck was cut in a standard way, and the location of the acetabulum was determined by following the inferior adhesion site of the capsule. Surrounding osteophytes and hypertrophic soft tissues were resected and cleared for a better view of the acetabulum. Starting with the smallest engraver, the acetabulum was engraved and prepared to ensure proper anteversion and inclination. The acetabular component was fixed to the true acetabulum by using an uncemented press fit and 2–3 titanium screws. The medulla of the femur was carved with carvings. Two points are placed on the proximal and distal parts in the same plane as the line along which the osteotomy was conducted. Transverse subtrochanteric osteotomy was performed from 1–2 cm distal to the minor trochanter. The proximal femur was sutured at 30 degrees retroversion of the proximal femur k line. Finally, the hip joint was reduced (Figure 1). The anteversion angle of the femur was increased in Group 1, unchanged in Group 2, and decreased in Group 3. The decision was made according to standard indications, radiological results, and intraoperative evaluation of patient anatomy.

The system used for THA was the Acetabular Cup System (Titanium Ti 6 Al 4 V Alloy) coated with a Titanium Porous Coating, and Bone Master was used in the study. Small acetabular cup system options (40–42 mm) can be used with a 22 mm Femoral Head. A titanium porous-coated femoral stem with a flat wedge design and a distal diameter between 6 mm and 20 mm was used to provide rotational and axial stability for the patient.

### 2.2. Clinical Evaluation

All patients were evaluated clinically and radiologically before surgery, once every three months during the first year, once every six months during the second year, and once every year thereafter until their last follow-up. Clinical evaluation consisted of physical examination and tests, along with radiological imaging. Sciatic and femoral nerve examinations were performed by physical examination with the utmost care, without electrophysiological tests. Range of motion (ROM) assessment was routinely performed and included hip flexion, extension, abduction, adduction, and internal and external rotation measurements. These values were recorded preoperatively and at each follow-up to evaluate functional recovery. Other physical examinations consisted of Q-angle measurement, Apley test, anterior and posterior drawer tests, McMurray test, pivot shift test, and varus and valgus stress tests. The Trendelenburg test determined abduction power. The length of each extremity was determined by measuring the distance between the anterosuperior iliac spine and the medial malleolus, and the subtraction of these values determined the difference between extremities. Several scoring systems were also used, including the AOFAS and Lysholm scores [8]. Radiological evaluation was performed preoperatively, postoperatively, and during follow-up with standard anteroposterior and lateral radiographs. The loosening of acetabular and femoral components was evaluated according to the system described by De Lee and Charnley and Gruen et al. [9,10]. The complications of (or after) surgery (dislocation, sciatic nerve damage, post-operative infection, malunion, and nonunion) were also evaluated and followed accordingly.

### 2.3. Postoperative Rehabilitation

All patients had undergone femoral shortening osteotomy; therefore, load bearing was introduced gradually. The patients were seated on their beds on post-op day 1. Earlier movements were started on post-op day 2, so the patient’s feet would only kindly touch the floor with a double crutch. In most patients, partial and complete loads were applied according to X-ray results in weeks 6 and 10. Patients with excessive soft tissue tension due to height extension were periodically put in the knee and hip flexion position for one-to-two weeks. The patient was deemed ready for a full load when full extension was achieved. Percutaneous iliotibial band relaxation was performed postoperatively in patients with genu valgus.

### 2.4. Statistical Analysis

The SPSS computer program (version 21.0) was used for all statistical analyses. Qualitative data were reported as numbers (n) and percentages (%) and were compared with the Chi-squared test. The normality of distribution was evaluated with the Shapiro–Wilk test. Parametric variables were reported as mean ± standard deviation (mean ± SD), comparisons among repeated measures were performed with the ANOVA and ANCOVA tests, and post-hoc tests were applied on variables that showed significant differences. Non-parametric variables were compared with the Kruskal–Wallis test for >2 group comparisons and the Mann–Whitney U test for 2-group comparisons. A *p*-value below 0.05 was accepted as statistically significant.

## 3. Results

In the current study, all patients underwent standard THA procedures with increased, unchanged, and reduced anteversion correction. There were 43 patients in Group 1 and their mean age was 50.2 (range, 34–67), there were 46 patients in Group 2 with a mean age of 54.2 (range, 37–65), and 41 patients were in the third group and mean age was 53.7 (range, 36–68) years. The mean follow-up duration was 49 months (min-max: 28–62 months). Regarding complications, 11 patients developed postoperative dislocation (Group 1: 3 patients, Group 2: 1 patient, Group 3: 7 patients, respectively). Apart from these, no early postoperative or late complications (sciatic nerve injury, postoperative infection, malunion, or nonunion) were observed.

Our study evaluated the effects of changes in anteversion angles (increased, unchanged, and decreased) on preoperative and postoperative clinical and radiological outcomes. The findings from Table 1, Table 2 and Table 3 are summarized below. The pre-op scores did not show significant differences among the groups regarding the AOFAS, Q-angle, and Lysholm scores (*p* > 0.05). Post-op scores demonstrated substantial differences among groups. The AOFAS score was significantly lower in Group 1 (84.49 ± 6.03) compared to Group 2 and Group 3 (92.74 ± 6.02 and 92.49 ± 6.45, respectively; *p* < 0.001). The Q-angle was the lowest in Group 3 (12.39 ± 2.58), with statistically significant differences among groups (*p* < 0.001). The Lysholm score significantly differed among the groups, with Group 1 scoring higher than Groups 2 and 3 (*p* < 0.001). The anteversion angle measured via CT was highest in Group 1 (53.05 ± 5.25) and lowest in Group 3 (24.56 ± 4.13), with statistically significant differences among groups (*p* < 0.001).

Pre-op results from Table 2 show no significant differences among groups in the Trip/stumble due to rotational problem, McMurray test, Apley test, Anterior drawer test, Posterior drawer test, Pivot shift test, Varus stress test, and Valgus stress test (*p* > 0.05). Post-op results indicate that Trip/stumble due to rotational deformity was most common in Group 1 (67.4%), whereas it was lowest in Group 2 (2.2%) and moderate in Group 3 (22.0%) (*p* < 0.001). The McMurray test was positive most frequently in Group 3 (31.7%) and least frequently in Group 1 (9.3%) (*p* = 0.031). Falling due to rotational deformity was highest in Group 1 (25.6%), while Groups 2 and 3 had significantly lower rates (*p* < 0.001). Dislocation after prosthesis was highest in Group 3 (17.1%), significantly different from Group 1 (7.0%) and Group 2 (2.2%) (*p* = 0.041). Aseptic loosening of implant components did not show a significant difference among groups (*p* = 0.602). Post-op ipsilateral knee pain VAS from Table 3 was highest in Group 1 (mean = 1.72) and lowest in Group 3 (mean = 0.39), with significant differences among groups (*p* < 0.001). Post-op contralateral knee pain VAS was similar in Groups 1 and 2 (0.628 and 0.630, respectively), whereas it was lowest in Group 3 (0.195) (*p* = 0.001).

Overall, increased anteversion was associated with poor post-op knee function, higher pain levels, and greater instability. Unchanged anteversion resulted in the best clinical outcomes. Decreased anteversion led to moderate functional improvements. These findings suggest that changes in anteversion angle significantly influence post-op function, pain, and stability, with increased anteversion negatively impacting clinical outcomes.

## 4. Discussion

Total hip arthroplasty (THA) in patients with adult developmental dysplasia of the hip (DDH) involves significant surgical challenges due to various biomechanical and anatomical changes. In patients with DDH, the disruption of the regular interaction between the femoral head and acetabulum significantly alters the physiology, neurovascular topography, and structure of the surrounding soft tissues of the hip joint [11]. Transverse subtrochanteric femoral shortening osteotomy is widely used, especially in patients requiring femoral anteversion correction. This technique increases prosthesis stability [12]. Degeneration and osteoarthritis may necessitate THA in patients with Crowe type 2, 3, and 4 high hip dislocations due to functional losses and pain [13,14]. In cases of high dislocation, the presence of acetabular and femoral deformities makes surgery technically tricky.

Femoral anteversion correction is performed using various methods during THA. Traditional methods aim to optimize anteversion by making incisions in the femoral shaft or by performing an osteotomy. Changchuan Li et al. reported that transverse subtrochanteric osteotomy provides better results than other anteversion correction methods [11]. Transverse subtrochanteric osteotomy was also applied in our study. In THA procedures performed without osteotomy, it is essential to maintain femoral leg length and not damage neurovascular structures. Higuchi et al. (2015) showed that leg lengthening of up to 5 cm was achieved with THA without osteotomy and that neurovascular complications were minimal [15]. However, this method has also been reported to increase the physical load on the reduction region and the risk of potential complications. Some studies have suggested that non-shortening THA can prevent length differences between legs and may also increase the healing rate; however, it may also increase physical strain on the reduction site. Yan et al. reported that the strain on the reduction site was associated with preoperative hip stiffness, and they indicated that this problem may be overcome by soft tissue release during the operation [16]. Similarly, we also performed capsule excision, iliopsoas tenotomy, and/or excision of tense bands in the gluteus medius to reduce strain on the reduction site when necessary. In addition, femoral anteversion correction may also affect postoperative gait mechanics. Incorrectly adjusted anteversion angle causes compensatory mechanisms to be activated in the hip and knee joints in gait analysis studies [17]. Therefore, the prosthesis placement angle should be carefully planned, and the leg length difference should also be considered.

In addition, long-term follow-up studies show that correct anteversion adjustment is critical for prosthesis durability and patient satisfaction [18]. Therefore, preoperative radiological evaluations of patients should be performed in detail, and optimal results should be targeted with individualized surgical planning. In this study, patients in whom the femoral anteversion angle was increased, left unchanged, or decreased were compared, and the effect of anteversion correction on clinical outcomes was evaluated. Increased anteversion was associated with postoperative knee dysfunction, high pain levels, and hip instability. Patients whose anteversion had not been changed showed the best clinical results. Moderate functional improvement was noted in patients with reduced anteversion angles. In addition, it was observed that the dislocation rates in the postoperative period were the lowest in Group 2, and this rate was recorded at the highest level, especially in Group 3 (*p* < 0.041). Alternative hip arthroplasty approaches, such as modular femoral stems or the combined anteversion technique, have been proposed to optimize component positioning and reduce dislocation risk [19]. However, our fixed-stem approach with controlled anteversion modification offers a cost-effective alternative with comparable clinical outcomes. Postoperative knee pain and instability were also seen the most in the increased anteversion group, and these findings were statistically significant (*p* < 0.001). In the postoperative period, changes in the anteversion angle were found to be determinant on the patients’ walking mechanics and general functional recovery levels. Changing the femoral anteversion angle significantly affected knee functions, pain levels, and hip stability in the postoperative period. These findings suggest that preserving the anteversion angle as much as possible during THA surgery may benefit [20]. However, longer-term and larger-sample studies on the subject are required.

This study is the first in the literature to compare the outcomes of different anteversion corrections during THA in adult DDH patients. Our results suggest that each approach has its advantages in terms of outcome. For example, patients with increased and unchanged anteversion had significantly fewer postoperative dislocations than those with decreased anteversion. In addition, patients with increased anteversion had higher Lysholm scores; however, they also had lower AOFAS scores and higher rates of falls and trips/stumbles due to rotational deformity. The difference between the AOFAS and Lysholm scores in the increased anteversion group may be because these two scores focus on different components. The Lysholm score focuses more on knee function, while the AOFAS score also includes hip-related elements such as pain and stability. Since pain and instability were more pronounced in the increased anteversion group, the AOFAS score showed lower results. These findings indicate that significantly changing anteversion (increase or decrease) may have more disadvantages than advantages. Therefore, it may be beneficial to prefer unchanged anteversion or try to limit the change as much as possible in THA surgery of untreated DDH patients.

### Strengths and Limitations

There are several limitations to our study. Firstly, patients’ relatively short follow-up time may be a limitation, as a more extended follow-up could have revealed later problems or recovery in patients. Although an average follow-up period of 49 months is sufficient for early and mid-term evaluations, more extended follow-up periods are needed to evaluate the implant’s long-term survival and late complications (loosening, osteolysis, etc.). Secondly, the study’s retrospective nature brings inherent limitations and may conceal possible bias during patient selection and surgery. Thirdly, we did not evaluate Merle D’Aubigne and Harris Hip Score in our patients. Lastly, the lack of a control group is a limitation; however, forming any control group among DDH patients with severe osteoarthritis (who require surgery to increase quality of life) would cause ethical problems. This is the first study in which the clinical outcomes of patients who received THA with different anteversion corrections are compared. Additionally, we calculated and evaluated many clinical parameters that may help determine research areas for future studies. We also observed that our sample size is relatively high compared to other THA/DDH studies. Therefore, despite its limitations, we believe it will shed light on future studies.

## 5. Conclusions

This study evaluates the effects of different anteversion corrections on clinical outcomes during total hip arthroplasty (THA) due to DDH. It shows that changes in the anteversion angle significantly affect postoperative knee function, pain levels, and hip stability. Our findings reveal that increasing anteversion led to worse clinical outcomes by increasing postoperative instability, knee pain, and the risk of falling/stumbling. In contrast, unchanged anteversion provided the best clinical outcomes, while decreased anteversion provided moderate functional improvements. These results suggest that preserving the anteversion angle as much as possible during THA may benefit surgical success and patient satisfaction. However, further large-scale, long-term studies are needed to confirm these findings.

## Figures and Tables

**Table 1:** Descriptive statistics and group comparisons of results (Continuous variables).

	Group 1 (n = 43)	Group 2 (n = 46)	Group 3 (n = 41)		
	Mean ± SD	Mean ± SD	Mean ± SD	F	*p* *
Pre-op scores					
AOFAS score	92.02 ± 6.39	92.33 ± 6.72	91.49 ± 7.95	0.16	0.855
Q-angle	13.6 ± 2.33	13.52 ± 2.21	14.49 ± 2.48	2.22	0.113
Lysholm score	82.88 ± 5.49	82.24 ± 4.85	81.15 ± 4.53	1.30	0.275
Post-op scores					
AOFAS score	84.49 ± 6.03 ^a^	92.74 ± 6.02 ^b^	92.49 ± 6.45 ^b^	49.39	<0.001
Q-angle	16.98 ± 4.24 ^a^	15.78 ± 3.63 ^a^	12.39 ± 2.58 ^b^	40.76	<0.001
Lysholm score	82.33 ± 4.01 ^a^	78.44 ± 4.03 ^b^	78.49 ± 5.02 ^b^	44.18	<0.001
Anteversion angle measured via CT	53.05 ± 5.25 ^a^	32.76 ± 4.91 ^b^	24.56 ± 4.13 ^c^	396.08	<0.001

* ANOVA, *p* < 0.05, Different superscript letters (a, b, c) in the same row indicate statistically significant differences between groups (*p* < 0.05). Groups sharing the same letter are not significantly different.

**Table 2:** Descriptive statistics and group comparisons of results (Nominal variables).

	Group 1 (n = 43) n * (%)	Group 2 (n = 46) n * (%)	Group 3 (n = 41) n * (%)	Chi-Square	*p*
Pre-op results					
Trip/stumble due to rotational problem	0 (0%)	0 (0%)	0 (0%)	N/A	N/A
McMurray test	6 (14.0%)	5 (10.9%)	5 (12.3%)	0.19	0.906
Apley test	4 (9.3%)	5 (10.9%)	1 (2.4%)	2.4	0.301
Anterior drawer test	6 (14.0%)	6 (13.0%)	4 (9.8%)	0.38	0.828
Posterior drawer test	2 (4.7%)	0 (0%)	0 (0%)	4.11	0.128
Pivot shift test	4 (9.3%)	5 (10.9%)	3 (7.3%)	0.33	0.849
Varus stress test	0 (0%)	0 (0%)	1 (2.4%)	2.19	0.335
Valgus stress test	0 (0%)	0 (0%)	0 (0%)	N/A	N/A
Post-op results					
Trip/stumble due to rotational deformity	29 (67.4%) ^a^	1 (2.2%) ^b^	9 (22.0%) ^c^	46.93	<0.001
McMurray test	4 (9.3%) ^a^	8 (17.4%) ^b^	13 (31.7%) ^c^	6.94	0.031
Apley test	4 (9.3%)	5 (10.9%)	6 (14.6%)	0.62	0.735
Anterior drawer test	9 (20.9%)	9 (19.6%)	12 (29.3%)	1.32	0.518
Posterior drawer test	2 (4.7%)	1 (2.2%)	0 (0.0%)	2.02	0.364
Pivot shift test	8 (18.6%)	9 (19.6%)	11 (26.8%)	1.01	0.605
Varus stress test	0 (0%)	0 (0%)	1 (2.4%)	2.19	0.335
Valgus stress test	8 (18.6%)	16 (34.8%)	12 (29.3%)	2.98	0.225
Falling due to rotational deformity	11 (25.6%) ^a^	0 (0.0%) ^b^	2 (4.9%) ^b^	17.91	<0.001
Dislocation after prosthesis	3 (7.0%) ^a^	1 (2.2%) ^a^	7 (17.1%) ^b^	6.40	0.041
Aseptic loosening of implant components	0 (0.0%)	1 (2.2%)	1 (2.4%)	1.01	0.602

* Number of positive results, Different superscript letters (a, b, c) in the same row indicate statistically significant differences between groups (*p* < 0.05). Groups sharing the same letter are not significantly different. N/A: Not Applicable, *p* < 0.05.

**Table 3:** Post-op ipsilateral and contralateral knee pain VAS scores and comparison of groups.

		Group 1 (Increased Anteversion) (n = 43)	Group 2 (Unchanged Anteversion) (n = 46)	Group 3 (Decreased Anteversion) (n = 41)	Chi-Square	*p* *
Post-op ipsilateral knee pain VAS	Mean	1.72	0.98	0.39	23.32	<0.001
	Median	2.00	0.50	0.00		
	Min	0.0	0.0	0.0		
	Max	4.0	4.0	2.0		
Post-op contralateral knee pain VAS	Mean	0.628	0.630	0.195	13.75	0.001
	Median	0.00	1.00	0.00		
	Min	0.0	0.0	0.0		
	Max	3.0	2.0	2.0		

* Kruskal–Wallis. *p* < 0.05.

## Data Availability

The data supporting this study’s findings are available from the corresponding author upon request.
